# Mobility data shows effectiveness of control strategies for COVID-19 in remote, sparse and diffuse populations

**DOI:** 10.3389/fepid.2023.1201810

**Published:** 2023-07-10

**Authors:** Yuval Berman, Shannon D. Algar, David M. Walker, Michael Small

**Affiliations:** ^1^ Complex Systems Group, Department of Mathematics and Statistics, University of Western Australia, Perth, WA, Australia; ^2^ CSIRO, Kensington, WA, Australia

**Keywords:** COVID-19, Google COVID-19 Aggregated Mobility Research Dataset, GAMRD, sparse population, compartmental model, Western Australia, transmission, remote

## Abstract

Data that is collected at the individual-level from mobile phones is typically aggregated to the population-level for privacy reasons. If we are interested in answering questions regarding the mean, or working with groups appropriately modeled by a continuum, then this data is immediately informative. However, coupling such data regarding a population to a model that requires information at the individual-level raises a number of complexities. This is the case if we aim to characterize human mobility and simulate the spatial and geographical spread of a disease by dealing in discrete, absolute numbers. In this work, we highlight the hurdles faced and outline how they can be overcome to effectively leverage the specific dataset: Google COVID-19 Aggregated Mobility Research Dataset (GAMRD). Using a case study of Western Australia, which has many sparsely populated regions with incomplete data, we firstly demonstrate how to overcome these challenges to approximate absolute flow of people around a transport network from the aggregated data. Overlaying this evolving mobility network with a compartmental model for disease that incorporated vaccination status we run simulations and draw meaningful conclusions about the spread of COVID-19 throughout the state without de-anonymizing the data. We can see that towns in the Pilbara region are highly vulnerable to an outbreak originating in Perth. Further, we show that regional restrictions on travel are not enough to stop the spread of the virus from reaching regional Western Australia. The methods explained in this paper can be therefore used to analyze disease outbreaks in similarly sparse populations. We demonstrate that using this data appropriately can be used to inform public health policies and have an impact in pandemic responses.

## 1. Introduction

In the age of mobile technology and the ever-increasing dependence on our electronic devices there is a complex interaction with inevitable data collection. The flow of data between a user and their smartphone is bidirectional. On the one hand, smartphones give access to a myriad of information we rely on as users. On the other hand, the individual’s personal data may be shared with apps for a variety of uses. For example, tracking of the user’s location can be enabled via their settings and used for related services such as traffic/weather updates or targeted advertising.

Mobile data collection also provides a means for the individual’s data to contribute to larger anonymized datasets, where aggregation of the individual-level data across space and time provides information about the broader population. For example, approximations of near real-time population-level mobility from individual GPS information (1).

The recent pandemic of COVID-19 caused an unprecedented shift in behavior as lockdowns and social isolation were employed to curb the spread of the disease. This is reflected in data that captures population movement over this time (2). Comparisons of the reduced movement patterns against pre-COVID baselines can enable us to properly quantify this change and determine the effectiveness of public messaging and large scale social distancing (3).

Despite these potential benefits, these datasets (often belonging to private companies) are typically not utilized to their fullest. In part, this can be attributed to the data’s “crypticness”, which results from having the appropriate legal, organizational and computational safeguards in place (4). This can be a significant first hurdle to using this data to drive or augment studies. Other hurdles include the sparseness of the data, as we will see in this paper, that can come from specific circumstances such as geographical remoteness.

We are interested in developing a more realistic epidemiologic model to explore how varying degrees of lockdown impact movement. To do so requires a proper understanding of movement, which can be achieved with these aggregated datasets. The primary aim of this paper is to outline the steps we took in order to make a particular dataset – The Google COVID-19 Aggregated Mobility Research Dataset (GAMRD) – compatible for such a task. We demonstrate how this dataset can be leveraged using Western Australia’s response to COVID-19 as a case study. Western Australia is unique both geographically and in how the pandemic was handled and while topical and close to home for the authors, this example was selected mostly because it captures many aspects of the challenges that can be faced when working with aggregated data. Specifically, we deal with sparse and small flows between rural towns with small populations, meaning our raw data is often incomplete, which necessitates a different approach to that of similar work done for densely populated regions (5).

To demonstrate the effectiveness of our methods in overcoming these issues we employ various data science techniques and a simulated compartmental model of disease dynamics to draw meaningful conclusions for the state of Western Australia. Specifically, we model the disease spread in Western Australia under three levels of movement: free movement, regional restrictions and full lockdown, exploring the impact on rural towns and the role that mining towns play in this spread. These constitute important, but secondary, aims of our work.

The paper is organized as follows. We first describe the dataset and outline how it has been used in studies thus far in Sections 2.1–2.2. We then demonstrate the steps taken to make this data useful when studying the movement patterns of a sparse population, as in Western Australia, in Sections 2.3–2.4. Section 2.5 gives an overview of COVID-19 in Western Australia, with relevant information pertaining to its geography, population and government responses. The compartmental model used to simulate the spread is described in Section 2.6 and in the Supplementary Materials. Our main results are in Section 3. The conclusions follow in Section 4.

## 2. Materials and methods

### 2.1. The google COVID-19 Aggregated Mobility Research Dataset (GAMRD)

The process below is all done by Google. The authors have only received the completed, anonymized version of the GAMRD in order to carry out our research.

The GAMRD contains anonymized mobility flows aggregated over users who have turned on the Location History setting, which is off by default. This is similar to the data used to show how busy certain types of places are in Google Maps—helping identify when a local business tends to be the most crowded. The dataset aggregates flows of people from region to region. This dataset has been used in several papers to study phenomena relating to human movement and mobility.

To produce the GAMRD, machine learning is applied to logs data to automatically segment it into semantic trips (6). To provide strong privacy guarantees, all trips were anonymized and aggregated using a differentially private mechanism (7) to aggregate flows over time (see https://policies.google.com/technologies/anonymization). This research is done on the resulting heavily aggregated and differentially private data. No individual user data was ever manually inspected, only heavily aggregated flows of large populations were handled. All anonymized trips are processed in aggregate to extract their origin and destination location and time. For example, if users traveled from location a to location b within time interval t, the corresponding cell (a,b,t) in the tensor would be n±η, where η is Laplacian noise. The automated Laplace mechanism adds random noise drawn from a zero mean Laplace distribution and yields (ϵ,δ) - differential privacy guarantee of ϵ=0.66 and $\delta =2.1\times 10^{-29}$ per metric. Specifically, for each week W and each location pair (A,B), we compute the number of unique users who took a trip from location A to location B during week W. To each of these metrics, we add Laplace noise from a zero-mean distribution of scale 1/0.66. We then remove all metrics for which the noisy number of users is lower than 100, following the process described in (7), and publish the rest. This yields that each metric we publish satisfies (ϵ,δ)-differential privacy with values defined above. The parameter ϵ controls the noise intensity in terms of its variance, while δ represents the deviation from pure ϵ-privacy. The closer they are to zero, the stronger the privacy guarantees.

These results should be interpreted in light of several important limitations. First, the Google mobility data is limited to smartphone users who have opted in to Google’s Location History feature, which is off by default. These data may not be representative of the population as whole, and furthermore their representativeness may vary by location. Importantly, these limited data are only viewed through the lens of differential privacy algorithms, specifically designed to protect user anonymity and obscure fine detail. Moreover, comparisons across rather than within locations are only descriptive since these regions can differ in substantial ways.

### 2.2. Uses of the GAMRD

The GAMRD has been used in many different ways in recent times, such as investigating relationships between socioeconomic status and movement patterns in the US and Brazil (8), or to develop a metric that establishes a connection between mobility indicators and key urban indicators relating to the livability of cities (6).

Regarding disease modeling, the GAMRD has been used to model spatial disease transmission and forecast influenza activity (9) to predict disease spread across state boundaries. This model performed on-par with models formed based on commuter surveys, which are less available and more expensive, investigating regions such as New York and New Jersey (dense population), and Australia (sparse population). Their study of Australia included an aggregation at the state level. In this work, we take this a step further and aggregate at a rural town level.

Aguilar et al. (10) used the GAMRD to assess the impact of urban structure on the spread of COVID-19, finding that hierarchical cities, where flows are concentrated primarily between mobility hotspots, are vulnerable to the rapid spread of epidemics, however mobility restrictions are very effective in mitigating the spread of the virus. Conversely, sprawled cities with many centers of activity experience slower spread, but response to mobility restrictions is less effective. Venter et al. (11) used the GAMRD to assess whether mobility in blue-green spaces (parks, natural areas) related to COVID-19 transmission, and found no such evidence. Ruktanonchai et al. (3) used the GAMRD to model COVID-19 transmission within Europe, specifically aiming to provide guidance on the best way to ease restrictions after the initial lockdown in Europe, finding that a large amount of coordination between countries is required to avoid a second spike of cases. Lemey et al. (12) similarly concluded that more effective and coordinated measures were required to contain the spread through cross-border travel, having used the GAMRD as part of their model of transmission of new variants in Europe.

### 2.3. Data preprocessing

The sparseness of the population in Western Australia has large implications on our model and the ability to draw conclusions from the GAMRD. In the GAMRD, cells are defined as 5.07 km${\;}^{2}$ regions across the entire globe, where movement by an individual (phone) between two different cells is recorded. As Western Australia’s population is sparse, there are times the GAMRD records zero movement between two towns that we would normally expect there to be movement between (more on how cells are assigned to towns below). Further, from a disease modelling perspective, the sparseness of the population in Western Australia meant that the disease spreads differently than the diffusion effect seen within cities or densely populated regions such as Europe (13). Therefore, in order to be able to use the GAMRD to draw meaningful conclusions on the spread of an infectious disease between towns in a population as sparse as Western Australia’s, several steps need to be taken to convert the data to a more usable form. These steps are generic and can for the most part be re-applied by other researchers looking to use the GAMRD in similar ways.

It is also important to note that these steps do not de-anonymize the information, due to the level of noise and other anonymization mechanisms included by Google in Section 2.1.

#### 2.3.1. Step 1: Assigning cells to towns

Firstly, we restrict the data to only include movement where the source and destination cells were inside Western Australia. After filtering out any cell outside Western Australia, we allocated cells to nearby towns, based on the geodesic distance between the cell and the town—so a cell is allocated to its closest town. This way, instead of having 5.07 km${\;}^{2}$ cells all around Western Australia, we define a network where each node is a town, and a connection between two nodes means movement has occurred between two towns over the course of the week. We selected the towns based on their population and location, with 44 towns across Western Australia (see Supplementary Material for full list of towns).

A cell was assigned to the closest town if that town center was at most 250 km away from the cell. If there are multiple town centers within 250 km of the cell, we assigned the cell to whichever town was closer. Setting an upper limit on the distance from town center meant that we inevitably lose some data, where one of the source cell or destination cell was not in the catchment area of any of the towns. The total amount lost was 0.881% of the total number of entries, a number we accepted. An upper limit of 250 km may appear large, however when considered in context of the vast distances in rural Western Australia,^1^ we decided it was an appropriate limit. A larger radius around each town may have captured more overall movement, however we risk having less clear boundaries between towns, particularly in areas more densely populated such as the South West. Smaller cutoffs could also be chosen, however these result in higher loss of data.^2^ Further, for cutoff values lower than 250 km, some mobility data from mining towns such as Newman, Port Hedland, Karratha, and Kalgoorlie-Boulder was discarded. These towns are particularly important in the context of Western Australia as they serve as regional hubs, with all of them having associated mine sites nearby. Therefore, we select 250 km as an effective compromise. In effect, the 250 km cutoff includes visitation to the nearest remote place as the data for a given town. In practice there is often movement from large population centers to locations that are up to 250 km (and more) from the nearest town. These reflect visits to individual settlements or parts of mine sites that are connected to the “nearby” remote center.

#### 2.3.2. Step 2: Adding a Kalman filter to deal with zero movement and noise

It is possible that zero movement may be recorded between two towns that we expect there to be movement between. This may be genuinely zero records or there may be too few records as the privacy algorithm stipulates a lower bound of 100 people after noise is added, with any number below being recorded as zero.

**Figure 1:**
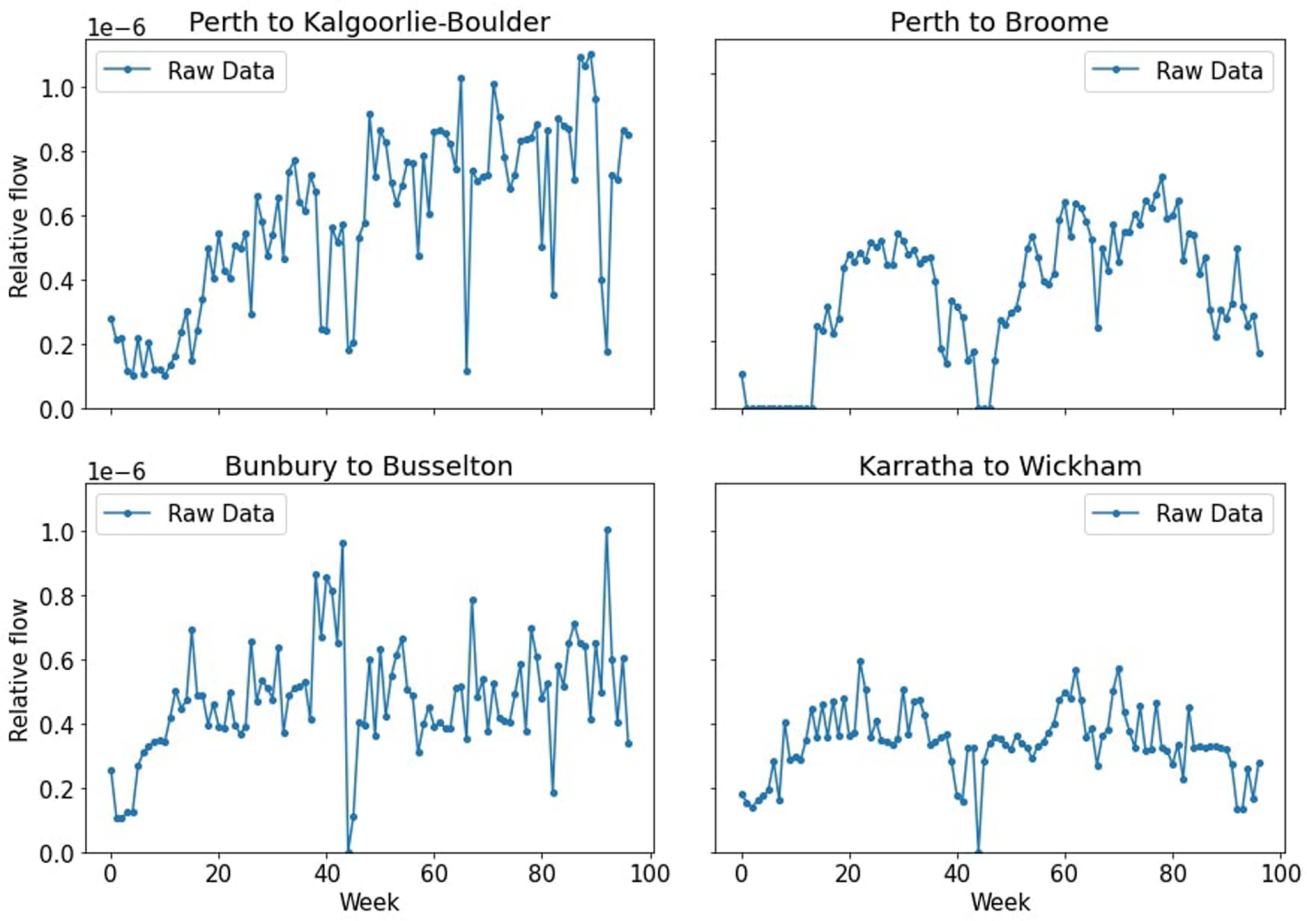
Relative flow of people between Western Australian towns. Note how the raw data can be quite noisy. For detail on the size and population of these towns, see Supplementary Material.

Further, although we could derive some interesting behavior on the macro-scale, we found that the GAMRD proved to be quite noisy on the town level—it may say there was no travel between two towns one week, before having a considerable spike in the very next week. This is shown in Figure 1, which displays the time series between four combinations of towns. This noisy data would cause problems when we look to use the GAMRD to infer which towns are vulnerable to the outbreak, as it may say there was no movement in and out of the town in a given week, even though we expect that to be false.

Due to this, we chose to apply a Kalman filter on all edges, which acted as a smoothing mechanism to better represent overall trends in how the movement between two towns changed over the time range of the data. We opted to use the Kalman filter over simpler techniques, such as a moving average, because the Kalman filter is able to model and estimate the underlying state of a system using probabilistic inference (14, 15). While the choice between a Kalman filter and a moving average is marginal (and discussed further in the Supplementary Material), the Kalman filter allows us to better integrate the limited temporal observations from sparse regions. The Kalman filter was defined by the following equations:

(1) $$\begin{array}{cc} \mu_{t} & =\mu_{t-1}+\Upsilon_{t}r^{-1}(y_{t}-\mu_{t-1}),\\ \text{where }\Upsilon_{t} & =((\Upsilon_{t-1}+qI{)}^{-1}+\frac{1}{r}I{)}^{-1}. \end{array}$$

Here, $\mu_{t}$ is the filtered value at week t, which is determined by the previous value plus a Kalman Gain times the *innovation*, $y_{t}-\mu_{t-1}$. The Kalman Gain term $\Upsilon_{t}r^{-1}$ is an adaptive term that also updates depending on its previous value and two noise terms, q and r, where q is the noise term of the model/estimate (where in our case, the model is just taking the previous term again) and r is the noise factor of the observations. Since we typically trust last week’s adjusted measurement ($\mu_{t-1}$) more than this week’s observation ($y_{t}$), we set r>q. This is due to the noisiness of this week’s observation; we decide to trust the dynamics we have set more than the raw data. Note that I is the identity matrix.

We set the initial values for $\mu_{0}∼N(\langle m\rangle ,\upsilon^{2}I_{N})$. This means that the initial estimate is approximated as the average weight of all edges, ⟨m⟩. For the initial covariance matrix, $\Upsilon_{0}$, we initialise it by finding the Pearson correlation coefficient between two edges. Hence the initialisation of the covariance matrix contained information on how the time series of one edge (e.g. Perth to Broome) related to another (e.g. Albany to Kalgoorlie). The reason this was chosen is because it approximated the initial transient of the time series better than when the covariance matrix was initialised as the identity matrix. After this initial transient, both choices for the covariance matrix lead to virtually identical time series. After experimentation, the parameters r and q were set to be 4∗d and 0.4∗d, where d is the mean of the initial Pearson correlation matrix. The aim of this was finding a combination of parameters that sufficiently smooth out the noise but still maintain the overall behavior.

**Figure 2:**
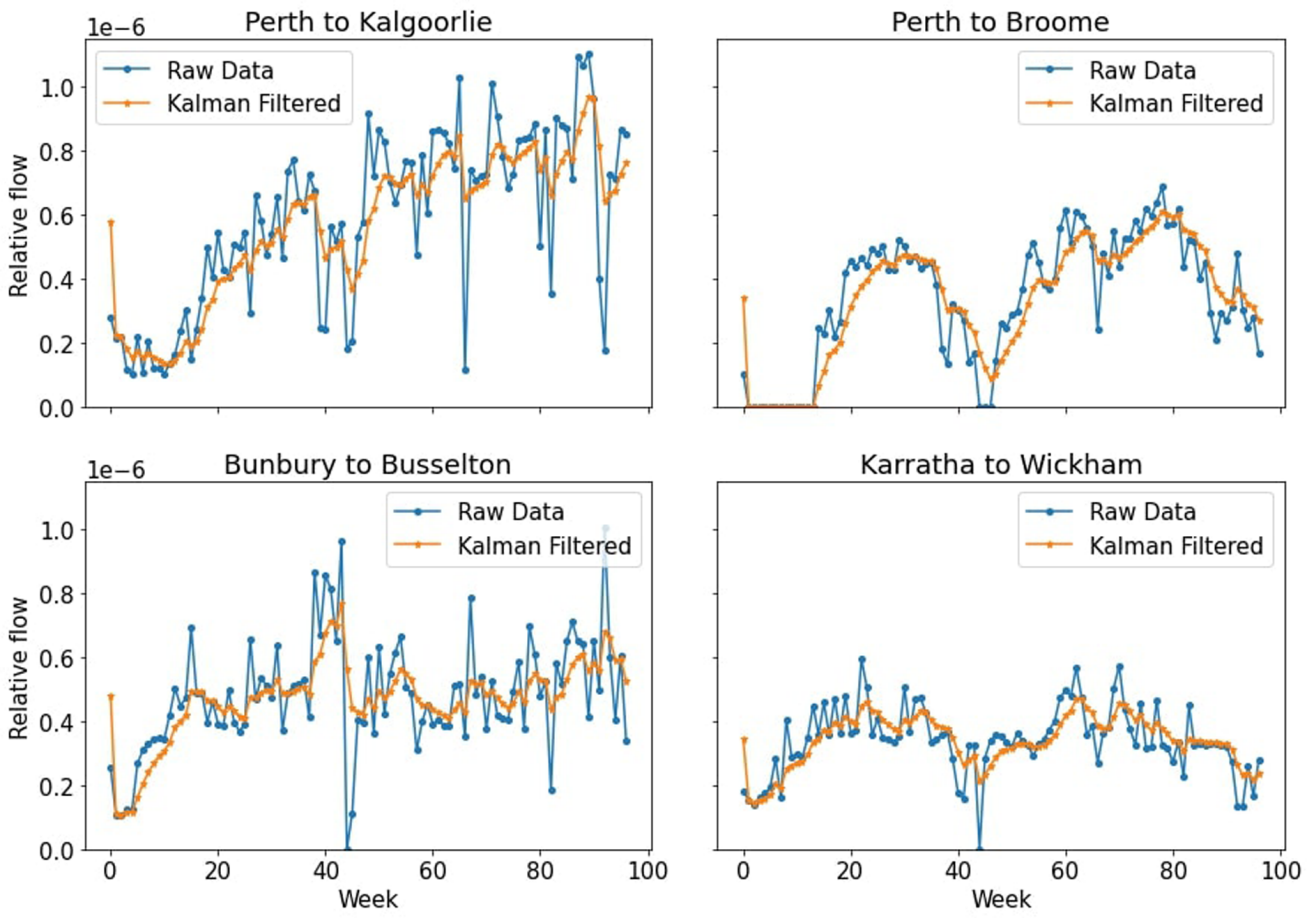
Results of the four in-focus time series after Kalman filtering was applied. The time series now have a smoother shape whilst still retaining the essential features of the overall behavior.

The results of the Kalman filter we applied on the four time series from Figure 1 can be seen in Figure 2. For more details on the Kalman filter and its comparison to a moving average, see the Supplementary Material.

#### 2.3.3. Step 3: Adding stochastic effects

We also chose to add stochastic effects to our networks in order to impute a daily resolution from the weekly movement data. This is due to the fact that in the GAMRD, even if a user travels from cell A to cell B multiple times in the same week, he or she only contributes once to the total count. Therefore, we add stochastic effects to the temporal adjacency matrices. We assumed that a user can move between two towns once every day, and examined how that affects the transport networks for the week. To do this we take the Kalman filtered adjacency matrix for each week, $A_{t}$; then normalize each row of the matrix to obtain the probability of moving to each town given the user is in a particular town; raise the transition matrix to the exponent of seven (one for each day of the week); add all matrices together $A_{t}+A_{t}^{2}+A_{t}^{3}+\cdots +A_{t}^{7}$ and divide them by seven to obtain the average. We then set the diagonal values to zero (we do not care for self-loops, as this is a transport network between towns), and convert the probabilities back to relative flows. Hence our adjacency matrices now include a stochastic element of increased movement. Heuristically, this stochastic element smooths the data by spreading out the movement around the different towns without artificially inflating the movement by adding new travelers.

**Figure 3:**
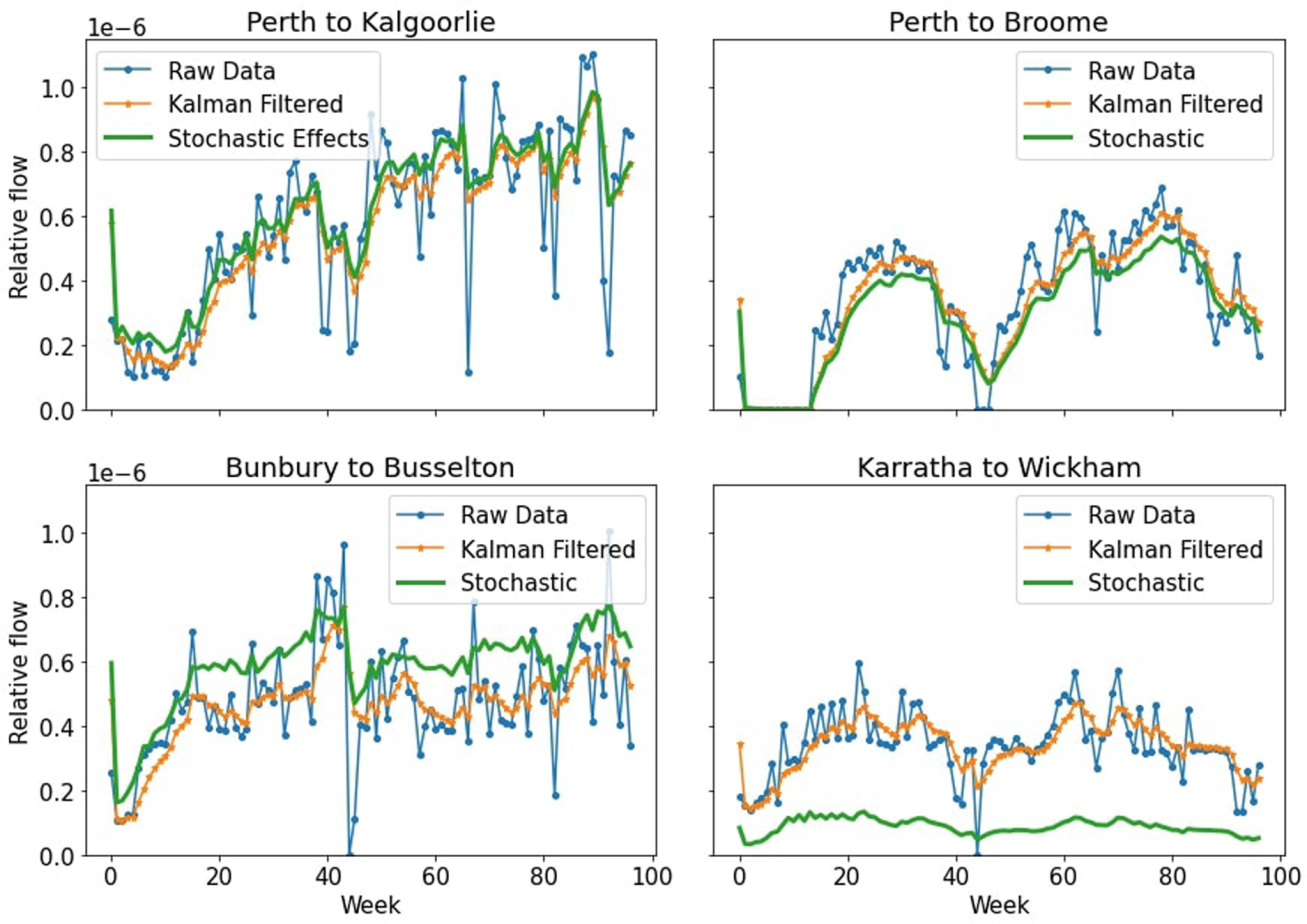
Results of the four in-focus time series after stochastic elements were added to each week.

In Figure 3 we can see the effects that adding the stochastic elements has had on the individual time series. Some time series increase their overall values, still maintaining their overall shape, while others decrease. This effect can be substantial, such as the Karratha to Wickham time series, however this can be explained by the fact that adding these stochastic effects actually adds edges that had not existed before, and this flow needs to come from other edges as we cannot add extra flow than what had existed previously. For an illustration of how the stochastic step changed the transport network for a given week, see the Supplementary Material.

#### 2.3.4. Step 4: Approximating absolute flow

**Figure 4:**
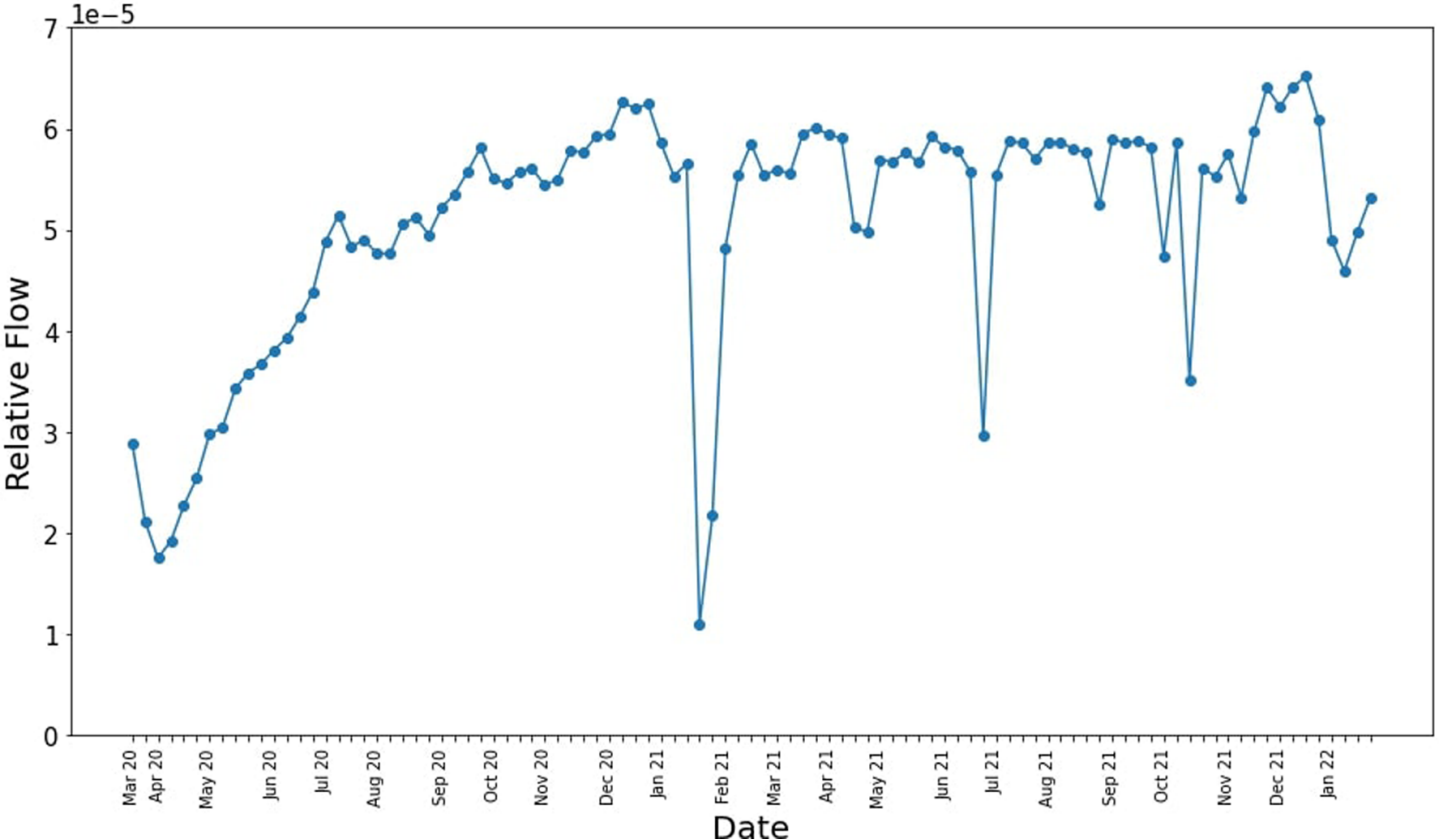
Relative total movement around Western Australia since the start of the pandemic.

The GAMRD provides us with some useful insights on the macro-scale. For example, analyzing the total movement around Western Australia in a given week since the start of the pandemic in Figure 4, we can see the initial low total amount of movement when the travel and regional movement restrictions were imposed in early 2020, and as restrictions slowly lift and people become more comfortable traveling within the state, the flow gradually increases. The two biggest dips occur when Western Australia had an immediate lockdown in response to a detected case of COVID-19; the first one occurring in Feb 2021 (16), and the second in late June to early July 2021 (17).

While the GAMRD is very useful for the *relative* flow of people between two towns, it is not so useful for the *absolute* flow between two towns that a model of disease transmission requires. Without absolute values describing how many people travel between two towns, we cannot deduce information such as when the disease reaches a new town.

Flows are aggregated temporally at a weekly level to obtain the GAMRD. This dataset contains normalized flows between pairs of cells in each week from March 22nd 2020 to January 29th, 2022. The flow is the natural logarithm of $\frac{U_{t,ij}}{F}$, where $U_{t,ij}$ is the number of unique users making a trip between cells i and j at week t, and F is an undisclosed constant (for privacy reasons) larger than the maximum flow over the entire year $F>\max_{t,i,j}U_{t,ij}$.

Disease modeling is a human subject; we deal in discrete, absolute number of human infections, discrete and absolute number of deaths, and a discrete absolute population in each town. We therefore need to know the probability that an individual who, for example, moves between town A and town B carries the disease with them, and hence we need to know how many individuals move between towns A and B each week in order to best estimate when the epidemic would reach town B. The relative flow is insufficient for this purpose; it tells us which towns are more closely connected to Perth, and hence may be more likely to get the virus if it originates in Perth, but it does not help us answer the critical question of *how many days* it will take for the virus to break into their towns. For this, we will need to have a better understanding of the absolute flow, which will require a more careful treatment of the value of the constant F. Note here that we are seeking to derive a model for movement that is representative of individual movement, but not of specific individuals.

We elected to do this by taking a known number of absolute travelers, obtained from the Australian Bureau of Statistics (ABS) (18) for a certain time period and comparing it to the relative flow according to the GAMRD for the same period. This allowed us to approximate a value for F, which will remain undisclosed. This method of reverse-engineering does not cause a privacy concern as it only provides a rough figure. The privacy of individuals is still protected using the differentially private algorithm and the lower bound of 100 people on recorded values being imposed.

### 2.4. Transport network results

**Figure 5:**
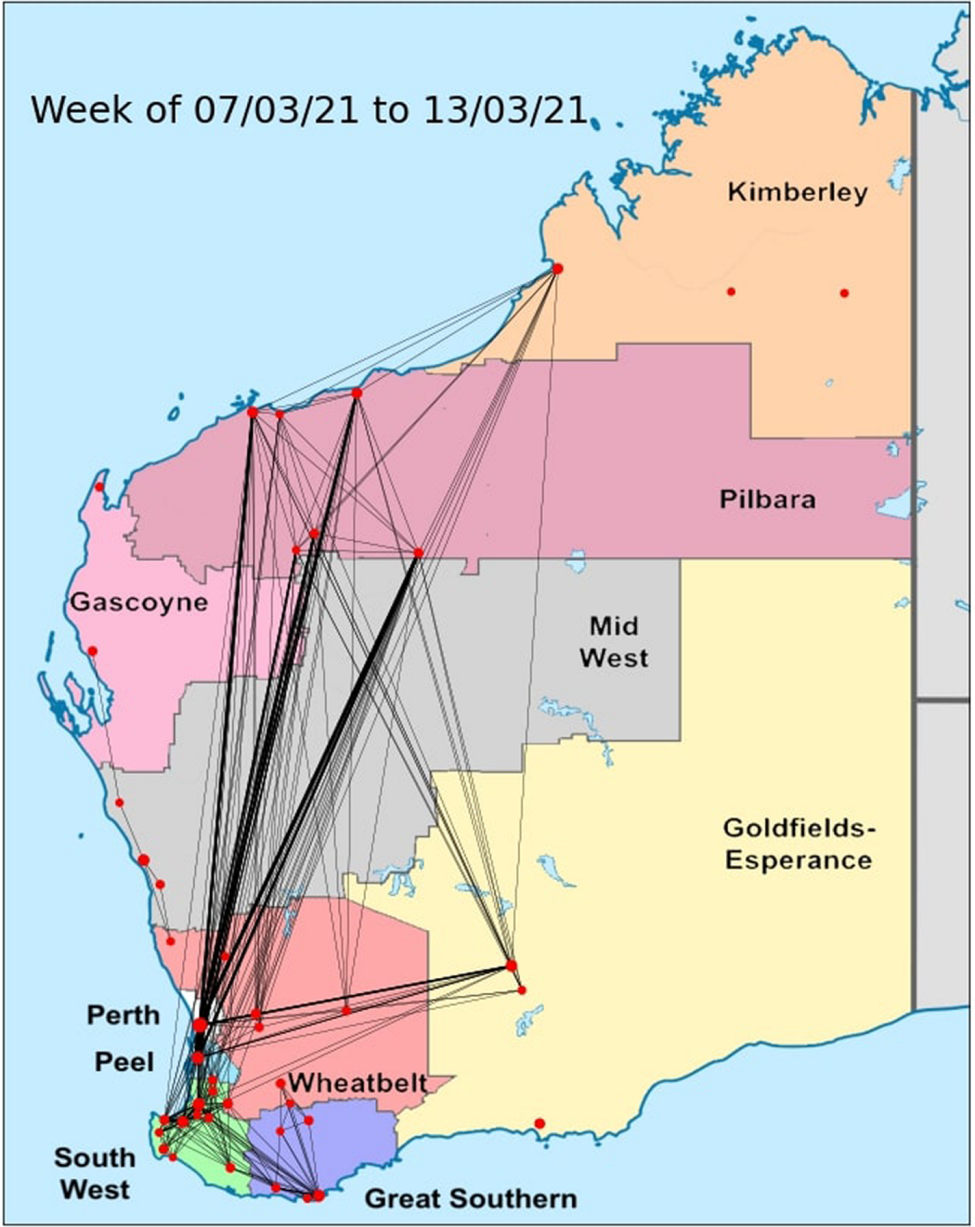
A movement network around Western Australia for a given week, generated from the GAMRD. Node size is correlated to population of the town, and edge thickness is correlated to the flow of people recorded within the specified week.

Having taken the steps above to convert the GAMRD to a more usable form for our purposes, we can now form temporal adjacency matrices. The nodes are the towns and the entries (edges) are the aggregated flow. These temporal adjacency matrices allow us to observe the evolution of the mobility network over time. An example of such a movement network over a week in Western Australia is presented in Figure 5.

Again, note that these steps do not de-anonymize the information, due to the level of noise and other anonymization mechanisms included by Google. However, now that the data is in this form, we can hope to obtain more realistic and valuable insights on the geographical spread of a disease in a sparsely located region such as Western Australia.

### 2.5. COVID-19 in Western Australia

Western Australia recorded its first confirmed case of COVID-19 on February 21st, 2020 (19). A State of Emergency was declared in Western Australia on March 15th, with a Public Health State of Emergency declared the next day (20). Gradual restrictions were introduced over the coming days by both the National Cabinet and the Western Australian government, with Western Australia banning regional travel on March 31st, and closing the interstate border on April 6th, 2020 (21). The last case of unknown^3^ community transmission of COVID-19 in Western Australia was recorded on April 11th 2020, and two weeks later restrictions slowly started being eased. However, the state border closure remained in place for far longer, with varying levels of Western Australia’s hard border^4^ in place for the next two years depending on the COVID-19 situation in other Australian states. This border would not come down for 697 days, until March 3rd, 2022 (22). For the majority of this time Western Australians experienced a relatively unaffected lifestyle within the state as no community transmissions were recorded. This was unique, as many states in Australia went through varying levels of outbreaks at different times during those two years. However, on December 21st, 2021, Western Australia recorded its first case of the Omicron strain of COVID-19 that is believed to have lead to the full outbreak experienced in Western Australia between from January 2022, despite early efforts to contain the virus as had been done previously (23, 24).

Western Australia’s remoteness, even when borders are open, is substantial; there are only two sealed roads leading in and out of the state that is 2.6 million km${\;}^{2}$ in size yet populated by just under 2.8 million people, leading to a population density of roughly one person per square kilometer. The majority of Western Australia’s population is located in the South West corner, with the capital city of Perth having a population of around 2 million people. The sparsity of population of Western Australia outside of Perth makes it an intriguing context in which to examine COVID-19 transmission. Knowing which towns are more susceptible to an outbreak that begins in Perth can inform governments on appropriate policies, and aid in making better informed decisions around implementing regional borders such as the one implemented on March 31st, 2020. Western Australia contains many remote Aboriginal communities who are more vulnerable to respiratory diseases such as COVID-19 (25). Hence, this information is of particular interest and government concern, as seen on February 20th, 2021, when a positive Omicron case was recorded in a remote Aboriginal community about 1,000 km east of Kalgoorlie (26).

**Figure 6:**
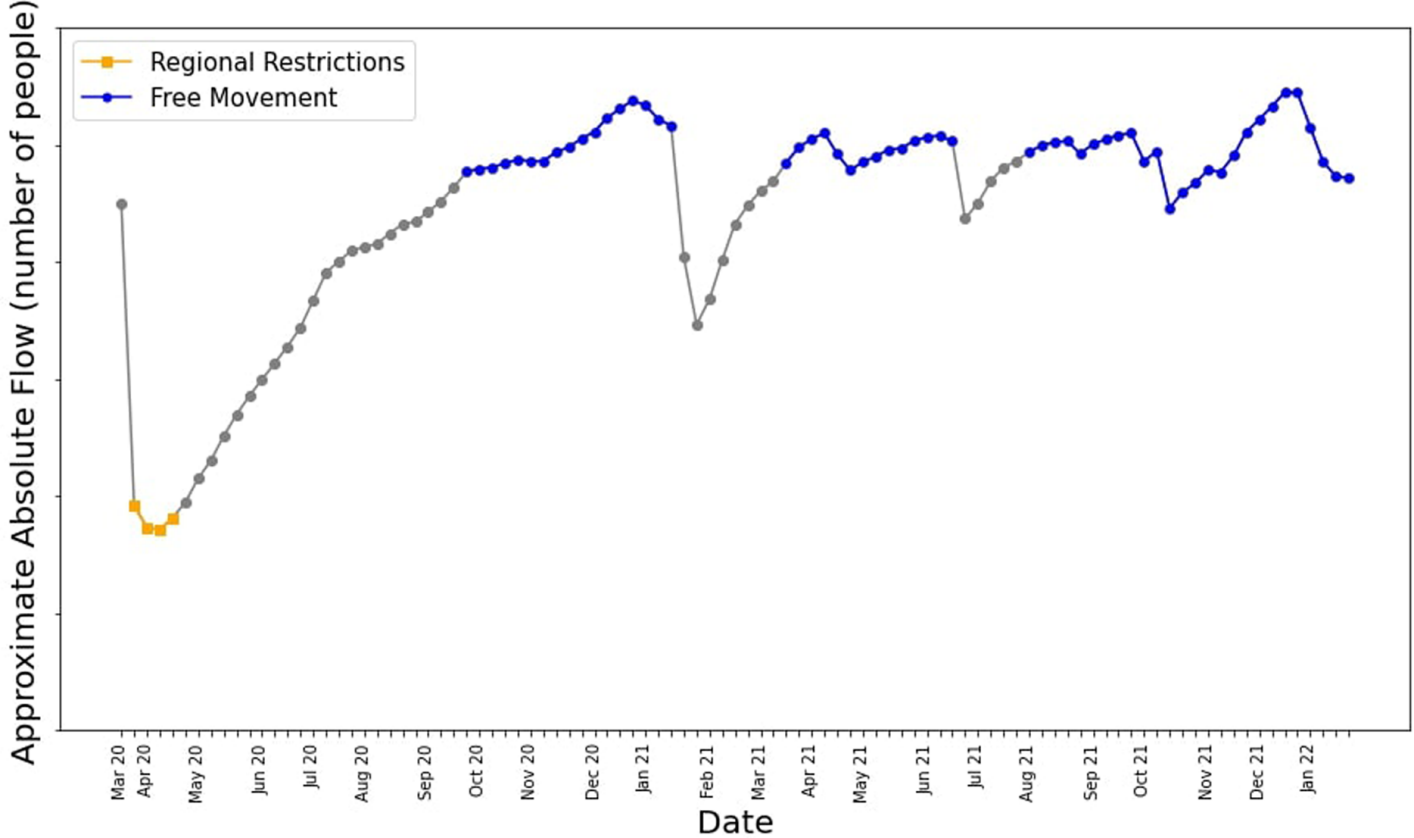
Total movement around Western Australia in a given week after the data preprocessing was carried out. Times when regional restrictions and free movement were in place are highlighted in yellow and blue respectively. Y-axis numbers were removed.

One of the key aims of this work was to quantify how the virus spreads under three levels of movement: *free movement*, *regional restrictions* and *full lockdown*. To quantify the differences between these three levels of movement, we compared the difference in movement between towns as is shown in Figure 6. We selected four weeks that correlate to when the initial lockdown occurred, and free movement during periods of relatively high movement, with some weeks left as grace period for movement to return to normal after a snap lockdown. Then, we quantified the difference to a full lockdown by analyzing the reduction in movement *within* towns during weeks of lockdown by analyzing the self loops of the initial transport networks we created. We used this reduction to modify the rate of infectivitiy, δ, in our compartmental model.

### 2.6. Compartmental model

We chose to use a compartmental model for disease spread within each town, as is often done when mathematically modelling COVID-19 in a homogeneous, fully-mixed population (5, 27, 28–30). Our model is based on the Susceptible-Exposed-Infected-Removed (SEIR) model, but the Susceptible (S) state has been split into the four different possible vaccination states at the time of carrying this modeling out: from unvaccinated ($V_{0}$) to triple vaccinated ($V_{3}$). Note that at the time of the work being carried out, no more than three doses were available to West Australians. However, the model can be easily modified to add more doses. Having separate vaccination compartments allowed us to gage the effectiveness of vaccines in slowing the spread of the disease. The compartmental disease model used in the paper are elaborated on in the Supplementary Material.

To determine the parameters that govern these equations, we first fit the model to the outbreak that happened in South Australia, which started a few weeks earlier than the one in Western Australia. As this work was done in the midst of these outbreaks occurring, we had to use the most recently available data, which initially was the South Australia outbreak. The rationale was that these states are similar in their population distribution and density. However, the parameters resulting from the South Australian outbreak did not do a good job of approximating the Western Australian one. This showed us that Western Australia and South Australia, as similar as they are in population density and distribution,^5^ are still different case studies. This shows the danger in using results from one outbreak to be generalized to another.

The parameters were instead fit to the Western Australian outbreak, which started a few weeks after the South Australian outbreak, and still whilst this work was carried out. The infectivity rate, δ, was a time sensitive parameter based on determined inflection points in the outbreak. The full parameter table and an explanation of the fitting methods is provided in the Supplementary Material.

#### 2.6.1. Making the compartment of an individual which moves between towns probabilistic

A deterministic compartmental model deals with continuous variables. However, the number of people and the population is discrete, not continuous. Hence, we need to make the number of people that move between each town, in each compartment of the model, discrete. Further, we want to add some probabilistic effects to the model to better reflect real movement—we want there to be some uncertainty in the number of people that move between towns in each compartment. To do this, we create a function that calculates the probability of an individual being in each compartment for town A. We iterate over the number of travelers between town A and town B, *randomly* assigning each traveler to a compartment based on the probability that an individual is in that compartment. This means there is some uncertainty in the way the model behaves, and adds a probabilistic nature that better reflects real world movement.

As an example, for town i in our 44 town model, the model equation for the unvaccinated ($V_{0}$) compartment, without taking mobility into account, is:

(2) $$\frac{\text{d}V_{0,i}}{\text{d}t}=-\frac{\delta V_{0,i}(t)I_{i}(t)}{N_{i}}-\mu_{1,i}(t).$$

Here, $N_{i}$ is the population of town i, and $\mu_{1,i}(t)$ is the number of people getting first dose vaccination^6^ at day t.

When we add the aspect of mobility between towns, Equation 2 becomes:

(3) $$\begin{array}{cc} \frac{\text{d}V_{0,i}}{\text{d}t} & =-\frac{\delta V_{0,i}(t)I_{i}(t)}{N_{i}}-\mu_{1,i}(t)+\sum_{j}(X_{\;ji}-X_{ij}),\\ \text{where } & X_{\;ji}∼\text{Bin}(A_{\;ji},\frac{V_{0,j}}{{\text{N}}_{j}}). \end{array}$$

The adjacency matrix of the mobility network is given by A, with entry $A_{\;ji}$ being the number of travelers that came into town i from town j. The probability of being in compartment $V_{0}$ in town j is the number of people there on the given day divided by the population of the town, i.e. $\frac{V_{0,j}}{N_{j}}$. All equations for our compartmental model for every town are similarly modified. See Supplementary Material for details.

**Figure 7:**
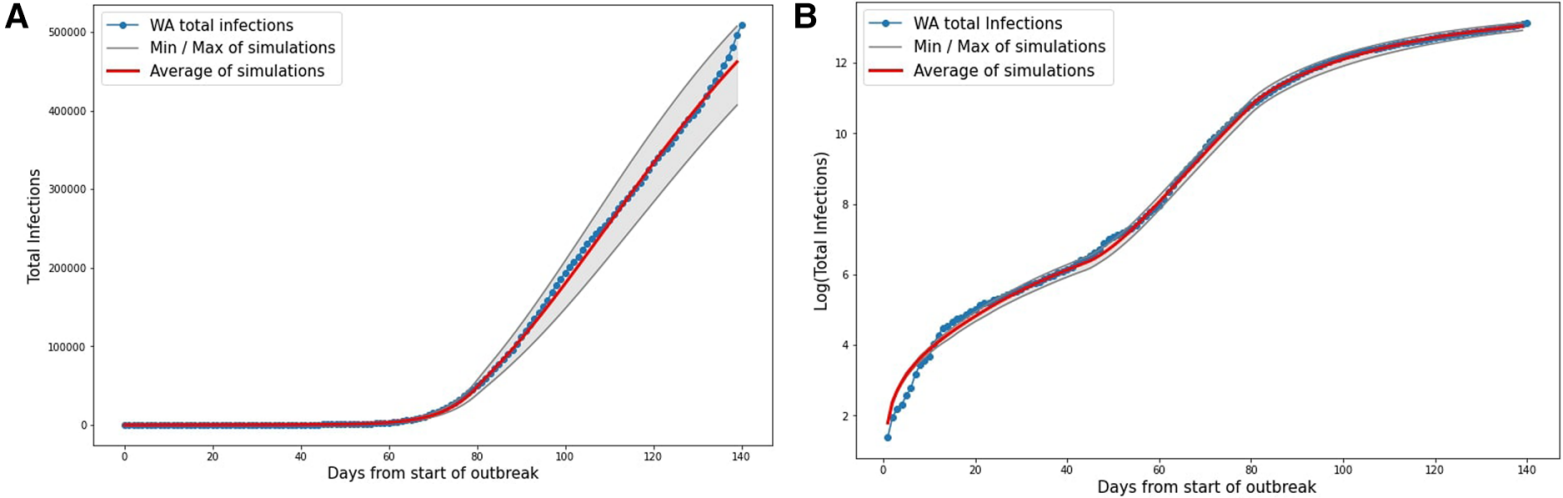
Results from 500 discrete model simulations with the derived parameters for the model. The gray shows the minimum and maximum of the 500 simulations, and the red line shows the average of all 500 simulations. (**A**) Total cases. (**B**) Logarithm of total cases.

The results of simulating the outbreak on a compartmental model in Western Australia with the transport network as described above can be seen in Figures 7A,B. Note that due to the probabilistic nature of our model, not all simulations result in exactly the same case numbers. However, we can see that the average does a good job of simulating the case numbers as they were reported.

**Figure 8:**
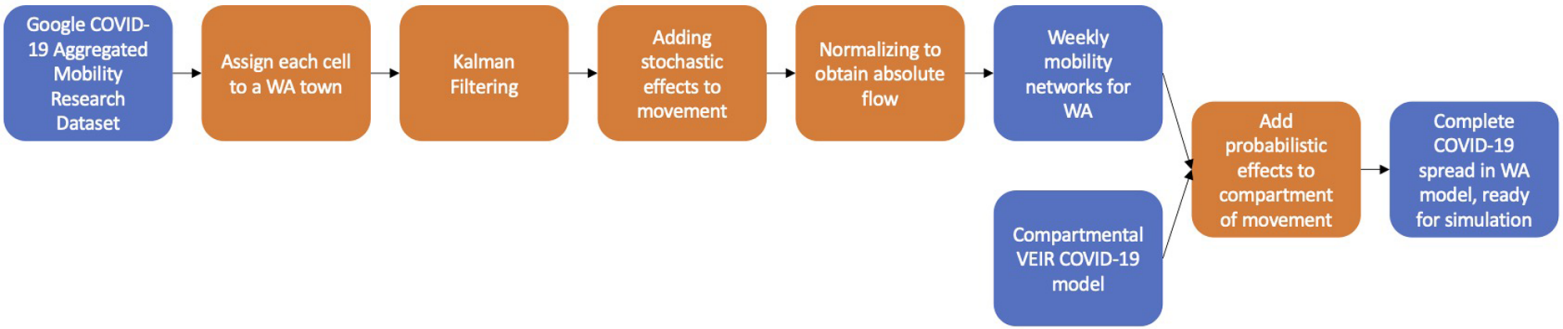
Pipeline of the process taken to create a model to simulate the spread of COVID-19 in a sparsely located population such as Western Australia. The blue represents input or output variables, while the orange represents manipulation in order to make the model be a better representation of reality.

To summarize, Figure 8 shows the entire process as a pipeline, from combining the GAMRD and a compartmental COVID-19 model in order to be able to simulate the spread of COVID-19 around Western Australia.

We have now created a successful model that can realistically simulate the spread of COVID-19 around a sparsely populated state such as that of Western Australia, based on the GAMRD and combined with a compartmental model of the Omicron strain of COVID-19. We can hence use this model to observe how the disease spreads around the state, which towns may be more vulnerable, and the effectiveness of different levels of restrictions on the spread of the disease.

## 3. Results

We run 10,000 simulations of an outbreak starting in Perth, with each level of movement around Western Australia (free movement, regional restrictions, full lockdown). We record the first occurrence of an infected individual reaching the town, simulating the outbreak for 180 days. The results of the simulations are shown in Table 1, with how often the outbreak reached the town within 180 days, and the average day this occurred. It also includes color coding by region (recall Figure 5), and is sorted by the average day for an infection to reach the town based on free movement.

**Table 1:** The percentage of simulations where an outbreak reached a town for the various restriction levels, and the average day the outbreak reached the town. Towns are colored by their region (refer to Figure 5 for a geographical view of the regions). The average day is calculated from the infections which did reach the town within 180 days, and is “NA” if no infections reached the town.

Region	Town	Free movement		Regional restrictions		Full lockdown	
		Infects town	Ave. day	Infects town	Ave. day	Infects town	Ave. day
Perth	Perth	100%	0	100%	0	100%	0
Peel	Mandurah	100%	7.8	100%	13.1	98%	25.2
Pilbara	Newman	100%	25.6	100%	33.5	49.9%	59.8
Pilbara	Karratha	100%	30.9	100%	43.4	25.5%	64.9
Pilbara	Tom Price	100%	34.5	100%	39.7	34.8%	64.2
Pilbara	Port Hedland	100%	35.6	100%	44.1	23.8%	67
Goldfields-Esperance	Kalgoorlie-Boulder	100%	36.1	100%	54.7	9.6%	70.8
Pilbara	Paraburdoo	100%	39.2	100%	50.5	14.9%	68.4
South West	Bunbury	100%	40.4	100%	66.1	3.8%	83.8
Kimberley	Broome	100%	41.3	100%	74.4	0.8%	78.5
Wheatbelt	Northam	100%	42	100%	63.9	4%	74.9
South West	Busselton	100%	45.8	100%	70.8	1.7%	85.4
Pilbara	Wickham	100%	46.5	100%	56.2	9.1%	71.4
Wheatbelt	York	100%	49.2	100%	65	3.8%	75.5
South West	Dunsborough	100%	51.7	100%	75.8	1.3%	87.4
South West	Donnybrook	100%	55.2	100%	78.6	0.8%	94
South West	Capel	100%	55.7	100%	106.7	0.2%	98.5
Goldfields-Esperance	Kambalda West	100%	56.8	100%	63.9	4.2%	74.5
South West	Collie	100%	57.9	100%	79	0.8%	86.9
South West	Cowaramup	100%	60.3	99.5%	119.4	0.2%	115.6
Wheatbelt	Merredin	100%	60.7	74.3%	110.5	0.1%	108.3
South West	Harvey	100%	61.1	100%	83.9	0.4%	101.1
Mid West	Geraldton	100%	64.7	100%	74.5	0.8%	70.2
South West	Manjimup	100%	65	84.2%	135	0%	158.3
Wheatbelt	Moora	100%	67.1	100%	84.4	0.4%	70.7
South West	Margaret River	100%	68.5	35.9%	145.8	0%	NA
Mid West	Port Denison-Dongara	100%	70.4	100%	84.2	0.4%	76.5
Wheatbelt	Jurien Bay	100%	76	95.9%	102.2	0.1%	29.8
South West	Augusta	100%	78	25.5%	147.3	0%	NA
Peel	Waroona	100%	78.9	100%	86.6	0.2%	58.3
Goldfields-Esperance	Esperance	100%	81.2	0%	NA	0%	NA
Great Southern	Albany	100%	82.2	0.4%	158.4	0%	NA
Wheatbelt	Narrogin	100%	84.6	0.2%	148.7	0%	NA
Great Southern	Denmark	100%	86.2	21.8%	143.8	0%	161
Gascoyne	Exmouth	100%	88.3	97.3%	108.6	0.2%	93.7
Great Southern	Kojonup	100%	90.2	2.4%	142.6	0%	NA
Great Southern	Little Grove	100%	90.9	0.2%	153.9	0%	NA
Great Southern	Katanning	100%	91.2	0%	127.2	0%	NA
Great Southern	Mount Barker	100%	97.5	0%	NA	0%	NA
Mid West	Kalbarri	99.9%	103.6	74.4%	110.9	0%	47.5
Gascoyne	Carnarvon	99.6%	116.1	99.8%	89.8	0.2%	71.2
Wheatbelt	Wagin	87.9%	130.3	0.1%	143.8	0%	NA
Kimberley	Halls Creek	0%	NA	0%	NA	0%	NA
Kimberley	Fitzroy Crossing	0%	NA	0%	NA	0%	NA

**Figure 9:**
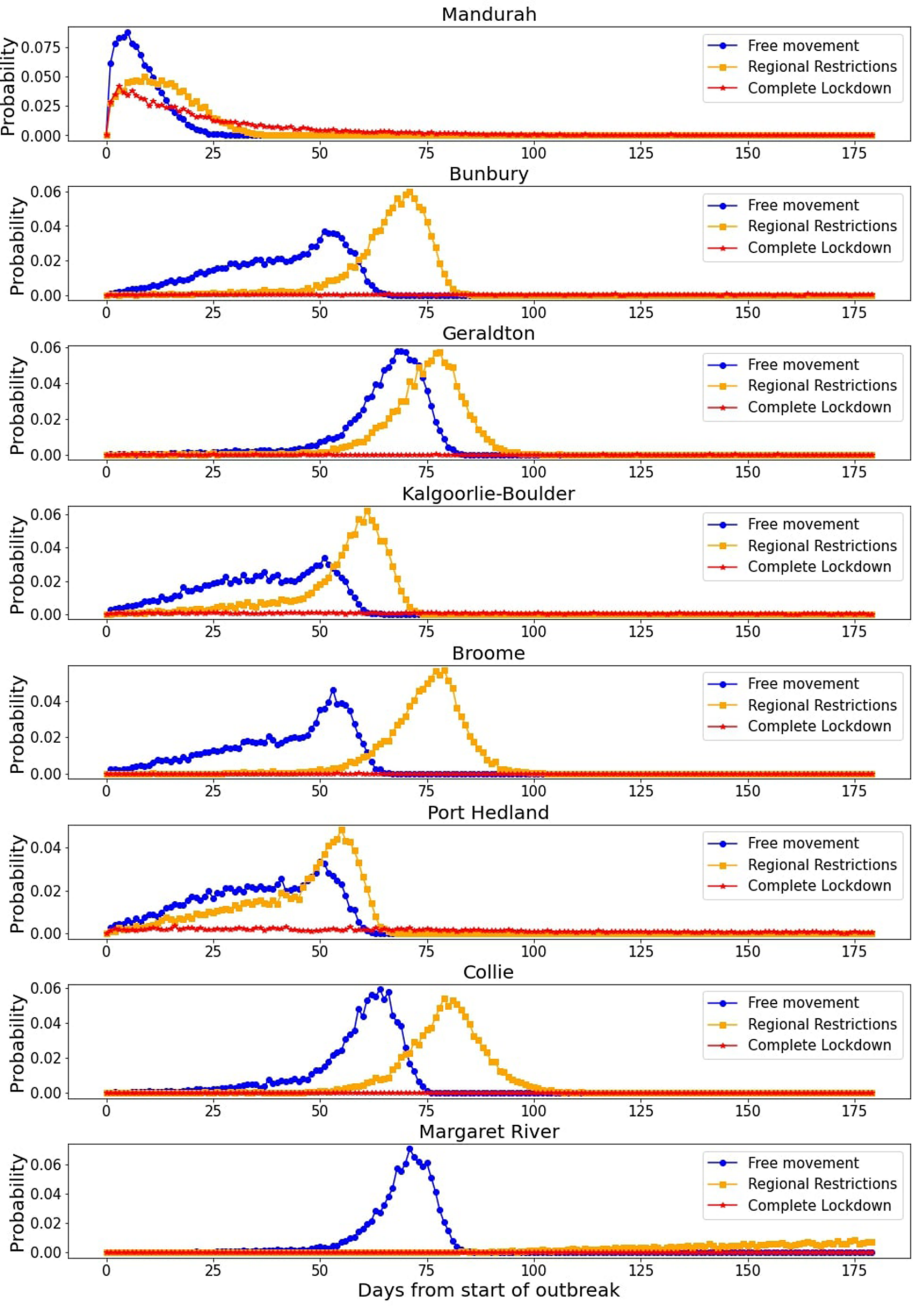
Time taken for an infectious case to reach the listed towns for the varying levels of restrictions. The x-axis represents days after the first infection is seeded in Perth, with the y-axis representing the probability of an infectious individual traveling to the town (or an exposed individual becoming infectious) on the given day after 10,000 simulations.

Figure 9 represents the probability that the outbreak reached a few select towns on a given day for the different levels of movement.

Analyzing Table 1 and Figure 9, we highlight a few key takeaways. Firstly, Mandurah is consistently the first town the outbreak reaches. This is hardly surprising, considering its proximity to Perth (the two are connected by a train line that consists of daily commuters). However, the next group of towns from Newman down to Paraburdoo are all mining towns, most of them in the Pilbara region. This is a key finding in the context of Western Australia—these towns have large nearby mines, which often operate on a FIFO (Fly-In-Fly-Out) roster. This means that large numbers of workers are flying between Perth and these mines, and were still flying in and out with regional restrictions in place. This finding highlights the role mining companies in Western Australia must play to keep the towns next to the mines they occupy safe. The towns near these mines are small (populations are in the thousands—see Supplementary Material) and often consist of communities vulnerable to the spread of COVID-19.

The total number of cases is in fact very well controlled by a full lockdown—total infections in the simulations do not break past the 300 mark even after six months. However, regional restrictions do not significantly lower case numbers—they simply protect rural towns for a bit longer.

We can see in Figure 9 how the different restriction levels impact the time taken for the outbreak to reach the towns in question. More stringent restrictions do slow the virus spread even for a town in close proximity to Perth such as Mandurah. However, the restrictions do not cause a major change in whether the outbreak will reach Mandurah, but rather it is a question of when. For the majority of other towns, regional restrictions increase the time taken, but the shape of the distribution remains largely the same. However, the complete lockdown does prove very effective, as we saw in Table 1, with all towns other than Mandurah very rarely experiencing an outbreak— this is due to the fact that a full lockdown was an effective method to suppress the spread in the simulation even in Perth.

## 4. Discussion

This work investigated utilizing the GAMRD to form a mobility network, which was used to draw meaningful conclusions about the spread of COVID-19 in Western Australia. The purpose of this work was to outline how many of the seemingly problematic features that are tied up with aggregated datasets such as the GAMRD can be overcome to derive a mobility model for discrete movement that is representative of a population, but still does not pose a threat to privacy of individuals. To build this network cells had to be assigned to towns, zero movement between town had to be considered and noisy data had to be smoothed, probabilistic effects had to be included and absolute flows had to be estimated. As an incredibly remote place, Western Australia exemplifies the issues with using this data in a sparse population and was hence used as a case study for the data.

The resulting network was successfully combined with a compartmental model for the Omicron variant of COVID-19, in order to simulate the spread of the disease around Western Australia. We have shown that this model is an effective and useful tool of modeling the spread of a disease geographically in a sparse population. We were able to derive insights about the vulnerability of towns to an outbreak starting in Perth, and therefore can use models such as this one to inform public health policy making. We showed regional restrictions had limited ability to slow the spread of the virus, however full lockdowns could still suppress the spread of the Omicron variant.

With the increase of mobile phone usage and consent-based data sharing, there is an increase in mobile phone data collection by private companies. This data will continue to exist and grow in size and detail. With the appropriate safeguards to preserve privacy in place the data may provide near real-time information and feedback about movement patterns. This is particularly relevant to the ongoing COVID-19 pandemic and helping to inform policy surrounding public health (4). Converting aggregated data to characterize individual-level movement need not de-anonymize the data or violate data protection goals in order to be informative. As we have shown, if properly handled, this data can be invaluable in developing effective controls to deal with disease outbreaks.

## Data Availability

The Australian National Statement on Ethical Conduct in Human Research requires ethical oversight at an institutional level for all research involving humans. According to the terms of reference of UWA Human Ethics Committee formal approval is required for research projects that generate new data. As the data used in this study was generated previously by Google, no further institutional approval was required. The Google COVID-19 Aggregated Mobility Research Dataset used for this study is available with permission from Google LLC.

## Footnotes

^1^For example, the distance between Kalgoorlie and the closest town in the dataset North of it, Newman, is almost 1,000 kilometers! Moreover, the distance between Margaret River in the South West corner and Kununurra in the North East corner of the state is almost 2,500 km!

^2^A 50 km cutoff causes a loss 6.47% of data, 100 km leads to 2.99% loss, 150 km leads to 1.51% loss, 200 km leads to 1.14% lost.

^3^The source of the infection was unknown.

^4^This hard border included international travel which was severely limited, and often included similar restrictions on travelers from other Australian states which had not controlled their outbreak.

^5^As of March 2022, there are approximately 2.8 million Western Australians at 1.0 persons per km${\;}^{2}$, versus 1.8 million South Australians at 1.7 persons per km${\;}^{2}$.

^6^Note that since town vaccination data is not readily available, we generalised the numbers from the state numbers.

## References

[1] Thums M Fernández-Gracia J Sequeira AMM Eguíluz VM Duarte CM Meekan MG. How big data fast tracked human mobility research, the lessons for animal movement ecology. Front Mar Sci. (2018) 5:21. 10.3389/fmars.2018.00021

[2] Campbell M Marek L Wiki J Hobbs M Sabel CE McCarthy J, et al. National movement patterns during the covid-19 pandemic in new zealand: the unexplored role of neighbourhood deprivation. J Epidemiol Commun Health. (2021) 75:903–5. 10.1136/jech-2020-216108 PMC8372376 33727245

[3] Ruktanonchai N Floyd J Lai S Ruktanonchai C Sadilek A Rente-Lourenco P, et al. Assessing the impact of coordinated COVID-19 exit strategies across Europe. Science. (2020) 369:1465–70. 10.1126/science.abc5096 32680881 PMC7402626

[4] Sills J Buckee CO Balsari S Chan J Crosas M Dominici F, et al. Aggregated mobility data could help fight COVID-19. Science. (2020) 368:145–6. 10.1126/science.abb8021 32205458

[5] Schoot Uiterkamp MH Gösgens M Heesterbeek H van der Hofstad R Litvak N. The role of inter-regional mobility in forecasting sars-cov-2 transmission. J R Soc Interface. (2022) 19:20220486. 10.1098/rsif.2022.0486 36043288 PMC9428544

[6] Bassolas A Barbosa-Filho H Dickinson B Dotiwalla X Eastham P Gallotti R, et al. Hierarchical organization of urban mobility and its connection with city livability. Nat Commun. (2019) 10:4817–10. 10.1038/s41467-019-12809-y 31645563 PMC6811587

[7] Wilson RJ Zhang CY Lam W Desfontaines D Simmons-Marengo D Gipson B. Differentially private SQL with bounded user contribution. Proc Privacy Enhanc Technol. (2020) 2020:230–50. 10.2478/popets-2020-0025

[8] Barbosa H Hazarie S Dickinson B Bassolas A Frank A Kautz H, et al. Uncovering the socioeconomic facets of human mobility. Sci Rep. (2021) 11:8616. 10.1038/s41598-021-87407-4 33883580 PMC8060260

[9] Venkatramanan S Sadilek A Fadikar A Barrett CL Biggerstaff M Chen J, et al. Forecasting influenza activity using machine-learned mobility map. Nat Commun. (2021) 12:726. 10.1038/s41467-021-21018-5 33563980 PMC7873234

[10] Aguilar J Bassolas A Ghoshal G Hazarie S Kirkley A Mazzoli M, et al. Impact of urban structure on infectious disease spreading. Sci Rep. (2022) 12:3816. 10.1038/s41598-022-06720-8 35264587 PMC8907266

[11] Venter ZS Sadilek A Stanton C Barton DN Aunan K Chowdhury S, et al. Mobility in blue-green spaces does not predict COVID-19 transmission: a global analysis. Int J Environ Res Public Health. (2021) 18:12567. 10.3390/ijerph182312567 34886291 PMC8656877

[12] Lemey P Ruktanonchai N Hong SL Colizza V Poletto C Van den Broeck F, et al. Untangling introductions and persistence in COVID-19 resurgence in Europe. Nature. (2021) 595:713–7. 10.1038/s41586-021-03754-2 34192736 PMC8324533

[13] Amdaoud M Arcuri G Levratto N. Are regions equal in adversity? A spatial analysis of spread and dynamics of COVID-19 in Europe. Eur J Health Econ. (2021) 22:629–42. 10.1007/s10198-021-01280-6 33751290 PMC7982906

[14] Walker DM Mees AI. Noise reduction of chaotic systems by Kalman filtering and by Shadowing. Int J Bifurcat Chaos. (1996) 7:769–79. 10.1142/S021812749700056X

[15] Anderson BDO, Optimal filtering. Dover books on electrical engineering. Newburyport: Dover Publications (2012).

[16] Department of the Premier and Cabinet. *Western Australia enters five-day lockdown from 6PM Tonight*. Government of Western Australia - Media Statements Website (2021). Available from: https://www.mediastatements.wa.gov.au/Pages/McGowan/2021/01/Western-Australia-enters-five-day-lockdown-from-6pm-tonight.aspx (Accessed April 26, 2022).

[17] Government of Western Australia. *4-day lockdown introduced for Perth and Peel* (2021). Available from: https://www.wa.gov.au/government/announcements/4-day-lockdown-introduced-perth-and-peel (Accessed April 26, 2022).

[18] Australian Bureau of Statistics. *Overseas travel statistics, provisional, June 2021* (2021). Available from: https://www.abs.gov.au/statistics/industry/tourism-and-transport/overseas-travel-statistics-provisional/latest-release (Accessed April 26, 2022).

[19] Borrello E. *First WA Coronavirus case flown to Perth for treatment after evacuating Diamond Princess ship* (2020). Available from: https://www.abc.net.au/news/2020-02-21/first-wa-coronavirus-case-to-be-flown-to-perth-for-treatment/11988920 (Accessed May 17, 2022).

[20] Department of the Premier and Cabinet. *COVID-19 coronavirus: state of emergency declarations* (2020). Available from: https://www.wa.gov.au/government/document-collections/covid-19-coronavirus-state-of-emergency-declarations (Accessed May 17, 2022).

[21] McNeill H. *A timeline of WA’s COVID-19 response: was our success luck, good management, or a bit of both?* (2020). Available from: https://www.watoday.com.au/national/western-australia/a-timeline-of-wa-s-covid-19-response-was-our-success-luck-good-management-or-a-bit-of-both-20200827-p55q03.html (Accessed May 17, 2022).

[22] Marcus L. *Western Australia ends one of the world’s longest border closures* (2022). Available from: https://edition.cnn.com/travel/article/western-australia-border-reopening-intl-hnk/index.html (Accessed May 1, 2022).

[23] Government of Western Australia. *WA Health. Media releases* (2021–2022). Available from: https://ww2.health.wa.gov.au/News/Media-releases-listing-page (Accessed March 26, 2022).

[24] Department of the Premier and Cabinet. *Public health and social measures introduced for Perth and Peel* (2021). Available from: https://www.wa.gov.au/government/announcements/public-health-and-social-measures-introduced-perth-and-peel (Accessed May 17, 2022).

[25] Fitts MS Russell D Mathew S Liddle Z Mulholland E Comerford C, et al. Remote health service vulnerabilities and responses to the COVID-19 pandemic. Aust J Rural Health. (2020) 28:613–7. 10.1111/ajr.12672 33216416 PMC7753557

[26] Hampton S. *COVID-19 outbreak declared at remote WA Aboriginal community Jameson or Mantamaru Community* (2022). Available from: https://www.perthnow.com.au/news/coronavirus/covid-19-outbreak-declared-at-remote-wa-aboriginal-community-jameson-or-mantamaru-community-c-5765419 (Accessed May 23, 2022).

[27] Small M Porras O Little M Cavanagh D Nicholas H. Modelling remote epidemic transmission in western australia and implications for pandemic response. *Unpublished* (2020).

[28] Leontitsis A Senok A Alsheikh-Ali A Al Nasser Y Loney T Alshamsi A. SEAHIR: a specialized compartmental model for COVID-19. Int J Environ Res Public Health. (2021) 18:2667. 10.3390/ijerph18052667 33800896 PMC7967501

[29] Carcione JM Santos JE Bagaini C Ba J. A simulation of a COVID-19 epidemic based on a deterministic SEIR model. Front Public Health. (2020) 8:230. 10.3389/fpubh.2020.00230 32574303 PMC7270399

[30] Grimm V Mengel F Schmidt M. Extensions of the SEIR model for the analysis of tailored social distancing and tracing approaches to cope with COVID-19. Sci Rep. (2021) 11:1–16. 10.1038/s41598-021-83540-2 33603113 PMC7893058

