# Supplementary material for: Mobility data shows effectiveness of control strategies for COVID-19 in remote, sparse and diffuse populations
Source: Front Epidemiol. 2023 Jul 10;3:1201810. doi: 10.3389/fepid.2023.1201810 (PMC10956099; doi:10.3389/fepid.2023.1201810)
Supplement: Supplementary file 1 [file Datasheet1.pdf]

frontiers

Supplementary Material

1 COMPARTMENTAL DISEASE MODEL

The compartmental disease model used in the paper can be seen schematically in Figure S1, with the arising equations shown in equations S1 to S9.

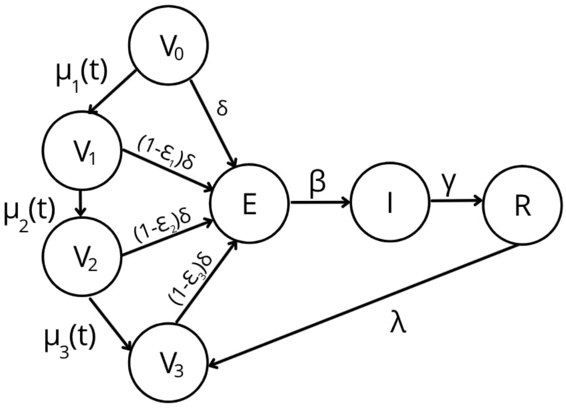

Figure S1. The chosen compartmental model for COVID-19 modeling in Western Australia.

The parameters can be explained as follows. The rate of infection is δ. The parameter that governs movement from the Exposed (E) to the Infected (I) state is β, which is related to the latent period of the disease: the time between an individual's infection and the onset of *infectiousness*[1]. The flow out of the I state depends on γ, which is the inverse of the period composed of the time the individual spends being infectious but asymptomatic plus the time he or she spends being symptomatic but not yet removed (such as after testing positive). People can move forward between the different vaccinated (V_m where $m = 0, 1, 2, 3$) states as they receive more doses of their vaccine, but not backwards. This movement is characterized by parameters $\mu_k(t)$, where $k = 1, 2, 3$, with $\mu_1(t)$ being the amount of people who received the first dose of the vaccine at day t. Further, the rate of infection parameter, δ, decreases proportionately to the effectiveness of each dose. We take ε_m as the efficacy of each dose, and the rate of infection parameter becomes $(1 - \varepsilon_m)\delta$ for each V_m state, where $\varepsilon_0 = 0$. Lastly, due to a waning immunity against the virus, there is a possibility for reinfection, albeit at a lower rate (Pulliam et al., 2021; Altarawneh et al., 2022; Turner, May 21, 2022). As such, we define λ as the parameter governing flow between the Removed (R) state and V_3. We set this as a connection to V_3 rather than V_0 as a recovered individual will still have some protection, and they will not be getting vaccinated again if they are already vaccinated, hence it will be misleading to put them in the Unvaccinated (V_0) state. Therefore, the equations for each town i are given below. Note we also add the mobility aspect to these single town equations, represented by the function $f(\cdot)$. This mobility component is described in the main text.

[1] It is important to distinguish here from the incubation period, which is the time between an individual's infection and the onset of *symptoms*

$$\frac{\mathrm{d}V_{0,i}}{\mathrm{d}t} = -\frac{\delta V_{0,i}(t)I_i(t)}{N_i} - \mu_{1,i}(t) + f(V_{0,i}), \tag{S1}$$

$$\frac{\mathrm{d}V_{1,i}}{\mathrm{d}t} = -\frac{(1-\varepsilon_1)\delta V_{1,i}(t)I_i(t)}{N_i} - \mu_{2,i}(t) + \mu_{1,i}(t) + f(V_{1,i}), \tag{S2}$$

$$\frac{\mathrm{d}V_{2,i}}{\mathrm{d}t} = -\frac{(1-\varepsilon_2)\delta V_{2,i}(t)I_i(t)}{N_i} - \mu_{3,i}(t) + \mu_{2,i}(t) + f(V_{2,i}), \tag{S3}$$

$$\frac{\mathrm{d}V_{3,i}}{\mathrm{d}t} = -\frac{(1-\varepsilon_3)\delta V_{3,i}(t)I_i(t)}{N_i} + \mu_{3,i}(t) + \lambda R_i(t) + f(V_{3,i}), \tag{S4}$$

$$\frac{\mathrm{d}E_i}{\mathrm{d}t} = \frac{\delta V_{0,i}(t)I_i(t)}{N_i} + \frac{(1-\varepsilon_1)\delta V_{1,i}(t)I_i(t)}{N_i} + \frac{(1-\varepsilon_2)\delta V_{2,i}(t)I_i(t)}{N_i} + \tag{S5}$$

$$\frac{(1-\varepsilon_3)\delta V_{3,i}(t)I_i(t)}{N_i} - \beta E_i(t) + f(E_i), \tag{S6}$$

$$\frac{\mathrm{d}I_i}{\mathrm{d}t} = \beta E_i(t) - \gamma I_i(t) + f(I_i), \tag{S7}$$

$$\frac{\mathrm{d}R_i}{\mathrm{d}t} = \gamma I_i(t) - \lambda R_i(t) + f(R_i), \tag{S8}$$

where $$f(X_i) = \sum_j \left(X_{ji} - X_{ij}\right) \text{, and } X_{ji} \sim \mathrm{Bin}\left(A_{ji}, \frac{V_{0,j}}{N_j}\right). \tag{S9}$$

Through a range of methods, including literature reviews and fitting Western Australia's outbreak data, we were able to derive values for each of the parameters, as shown in Table S1 (Berman, May 27, 2022).

Table S1. Best fit parameters for the first 141 days of the Western Australian Omicron outbreak, which is the limit put on when this work was carried out.

Parameter	Time Period	Value	Method
$\mu_k(t)$	Variable	Variable	Based on daily vaccinations (Covid Live, 2022)
β	$\forall t$	1/2.4	Literature review (Zhao et al., 2021; Xin et al., 2021; World Health Organisation, February 17, 2022)
λ	$\forall t$	1/84	WA government's information on immunity provided after COVID infection (Department of Health, Government of Western Australia, May 6, 2022)
ε_1	$\forall t$	0.293	Vaccination efficacy review (Andrews et al., 2022)
ε_2	$\forall t$	0.126	Vaccination efficacy review (Andrews et al., 2022)
ε_3	$\forall t$	0.566	Vaccination efficacy review (Andrews et al., 2022)
γ	$\forall t$	1/3.778	Fitting South Australia outbreak
δ_1	$0 \leq t < 16$	0.435	Fitting Western Australia outbreak
δ_2	$16 \leq t < 44$	0.448	Fitting Western Australia outbreak
δ_3	$44 \leq t < 80$	0.897	Fitting Western Australia outbreak
δ_4	$80 \leq t \leq 141$	0.560	Fitting Western Australia outbreak

To work out ε_m, we used the vaccine efficacy presented in (Andrews et al., 2022), and derived a general value for each dose based on the proportion of different vaccine types (e.g. AstraZeneca, Pfizer, Moderna) available in Australia at the time of writing. Since γ is the time taken before an individual is *removed* from the population rather than *recovered*, we could not rely on the recovery time for COVID-19 as reported in the literature. Therefore, we derived γ by fitting the compartmental model using SciPy's `curve_fit` function (Virtanen et al., 2020) to the daily infectious cases of the Omicron outbreak in South Australia. As this work was done in the midst of these outbreaks occurring, we had to use the most recently available data, which initially was the South Australia outbreak. The rationale was that these states are similar in their population distribution and density. When estimating δ, we opted for a similar method. However, this did not translate well between the states, meaning the compartmental model did not do a good job of estimating Western Australia's case numbers. This showed us that Western Australia and South Australia, as similar as they are in population density and distribution[2], are still different case studies. This illustrates the danger in using results from one outbreak to be generalized to another. Therefore, we approximated δ using SciPy's `curve_fit` and fitting the model to the WA outbreak, attributing it as a time sensitive parameter due to the different behaviour of the virus at different stages of the outbreak. We take this as reasonable act as the aim of the paper was not to estimate the rate of infection in Western Australia, but rather show the validity of applying the GAMRD to a sparse population, and show how, when combined with a compartmental model, it can make meaningful conclusions about COVID-19 in Western Australia.

2 KALMAN FILTERING AND ADDING STOCHASTIC EFFECTS

2.1 Kalman filtering vs. a moving average

In our work, we chose to use a Kalman filter as the smoothing mechanism for our noisy time series data of movement between two towns in a given week. We opted to use the Kalman filter over simpler techniques, such as a moving average, because the Kalman filter is able to model and estimate the underlying state of a system using probabilistic inference (Walker and Mees, 1996; Anderson, 2012). Further, the Kalman filter can effectively estimate the true underlying value even in the presence of noisy observations. Conversely, a moving average is a simple smoothing technique that calculates the average of a subset of data points over a fixed window. It can reduce high-frequency noise but may not handle complex noise patterns as effectively as the Kalman filter. Due to the level of complexity present in the transport network, we chose to use the Kalman filter.

With a Kalman filter, we could also investigate whether adding the relationship between edges to the Kalman filter improved the model. To do this, we initialised the covariance matrix (Υ_0) as the Pearson correlation coefficient between two edges. Hence, the covariance matrix contained information on how the time series of one edge (e.g. Perth to Broome) related to that of another (e.g. Albany to Kalgoorlie). After a few time steps, this model quickly approximated the result of applying the Kalman filter where the covariance matrix was initialised as the identity matrix. However, the key difference was that the correlation coefficient initialisation did much better at approximating the initial transient, and so it was chosen over the identity matrix initialisation.

Figure S2 shows the difference between the different initialisations of Υ_0, as well as a comparison to a five-step moving average. By examining the figure, we can see the Kalman filter is not as affected by noise. Further, since it takes into account the dynamics of the system we can see it often holds a pattern

[2] As of March 2022, there are approximately 2.8 million Western Australians at 1.0 persons per km^2, versus 1.8 million South Australians at 1.7 persons per km^2

of behaviour for longer (such as going up more slowly after the initial dip in movement). Pragmatically, any of the methods illustrated would have been reasonable choices to smooth this data. However, for the reasons mentioned above, we chose to employ the Kalman filter with the covariance matrix initialised as the Pearson correlation matrix.

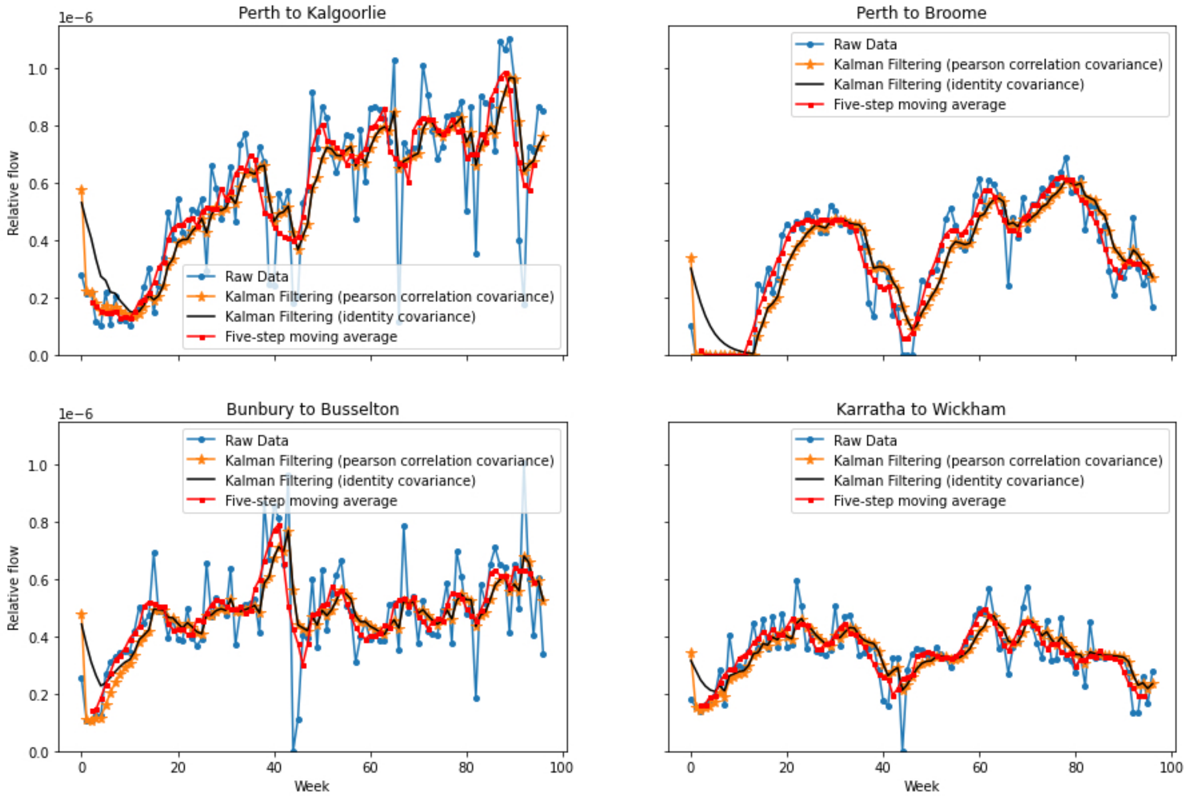

Figure S2. A comparison of how different methods perform in smoothing out the time series. We can see they are all relatively similar but have some small differences.

2.2 Transport network before and after adding stochastic effects

It is perhaps easiest to appreciate the full effect of the stochastic step on a visual transport network for a given week. Figure 3 shows the transport network in Western Australia before and after adding the stochastic step. We can see that this paints a much more realistic picture of travel in a given week. Therefore, this achieved our desired goal of smoothing the data by spreading out the movement around the different towns without artificially inflating the movement by adding new travelers.

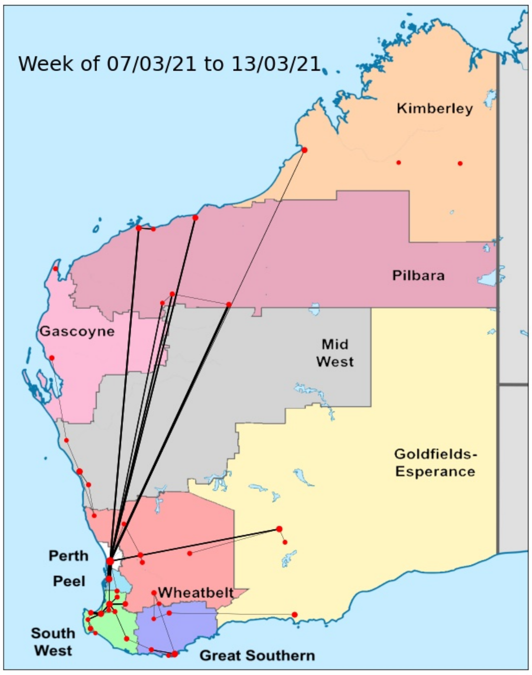

Figure 3a. Transport network prior to adding stochastic effects for a given week.

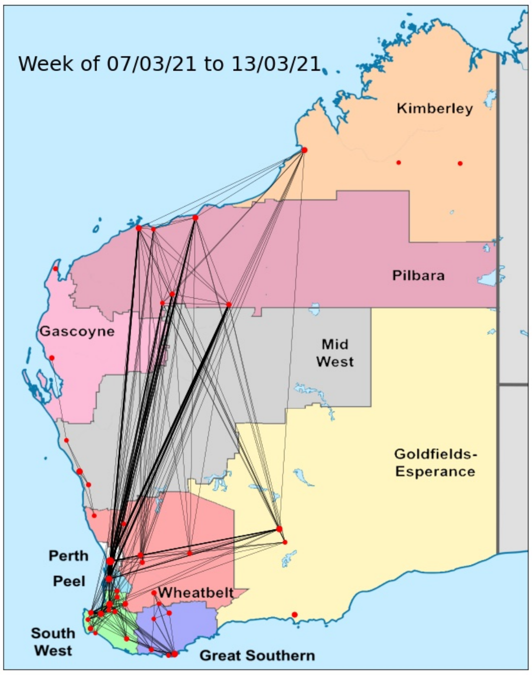

Figure 3b. Transport network after adding stochastic effects for a given week.

Figure 3. Comparing the transport network before and after the stochastic step. Note the added edges that are now present in the network.

3 USING EFFECTIVE REPRODUCTION NUMBER TO ESTIMATE VACCINATION LEVELS REQUIRED TO SUPPRESS OUTBREAK

On January 21st 2022, the Western Australian government reversed its decision to open the Western Australian hard border on February 5th (Kagi, January 21, 2022). The reasons cited for this was the emergence of the Omicron strain, and the concern that two vaccine doses offered limited protection against catching Omicron. Hence, it was hoped that the government's delayed opening would allow for an increase in third dose vaccination rates.

With the emergence of Omicron in Western Australia and the inability to suppress it as had been done previously with other strains, the government ultimately opened the border a month later, on March 3rd (Marcus, March 3, 2022). We want to estimate the booster (third dose) vaccination rate that would have been required for Western Australia to effectively suppress the virus, had it been kept out.

To do this, we consider the reproduction number:

$$R_0 = \frac{\delta S_0}{\gamma}, \tag{S10}$$

where S_0 is the proportion of individuals initially in the Susceptible compartment, and γ is the recovery rate. The meaning of the reproduction number is that it is the average number of individuals that an infected person infects. If $R_0 > 1$, an epidemic ensues. If $R_0 < 1$, the disease dies out.

However, our model presents four susceptible states, rather than one, with different size populations and different (yet related) rates of infection parameters. Further, after some time t we can have people in the Removed state, where they cannot be infected with the virus. Hence, we rename the basic reproduction number the *effective* reproduction number (Delamater et al., 2019), as by definition it does not assume a complete susceptibility of the population. We want an effective reproduction number $R_{\text{eff}}(t)$ that can adapt based on time t and takes into account the four different susceptible states (one for each vaccination level) and their rates of infection. Hence we firstly build a weighted, time dependent transmissibility parameter that encompasses all four susceptible states and their effective transmissibility parameters. We also want to know the proportion of people in the four Susceptible states combined, which we define as $S_0(t)$. Therefore, we define $R_{\text{eff}}(t)$ for our model:

$$R_{\text{eff}}(t) = \frac{\delta}{\gamma}\left(\sum_{m=0}^{3}(1-\varepsilon_m)\hat{V}_m(t)\right)\left(\sum_{m=0}^{3}\hat{V}_m(t)\right).$$

Here, ε_m is the effectiveness of each dose of the vaccine, and $\hat{V}_m(t)$ represents the portion of the population in each of the four susceptible compartments.

We assume that we wish to suppress an outbreak that has not yet spread, meaning that effectively all of Western Australia's population is in one of the four susceptible states. Hence $\sum_{m=0}^{3}\hat{V}_m(t) = 1$.

We first look at an analytic solution that assumes an individual is either completely unvaccinated or triple vaccinated. This will help us find an upper limit for the percentage of triple vaccinated people required to

keep $R_{\text{eff}}(t) < 1$. Hence, setting $V_1, V_2 = 0$, $V_0 + V_3 = 1$, and setting $R_{\text{eff}}(t) < 1$, the equation becomes:

$$1 > \frac{\delta}{\gamma}\Big(\hat{V}_0 + (1-\varepsilon_3)\hat{V}_3\Big),$$
$$\implies \frac{\delta}{\gamma} > 1 - \varepsilon_3\hat{V}_3,$$
$$\implies V_3 > \frac{1-\gamma/\delta}{\varepsilon_3}.$$

Earlier, we showed that $\gamma = 1/3.778$ and $\varepsilon_3 = 0.566$, and we found that $\delta_0 = 0.442, \delta_1 = 0.455, \delta_2 = 0.905, \delta_3 = 0.567$. We hence find the required proportion of people to be triple vaccinated for each phase of the outbreak in Table S2.

Table S2. Required percentage of triple vaccinated people in Western Australia to suppress the spread of Omicron based on the different infectivity rates (δ) found for the four different phases of the outbreak, starting at days 0, 16, 44 and 80 since the first case was detected.

Start of Period	δ	$\hat{V}_3$
$t = 0$	0.442	70.9%
$t = 16$	0.455	73.9%
$t = 44$	0.905	125%
$t = 80$	0.567	94.2%

The first two rows of Table S2 show that only 71% and 74% of people had to be triple vaccinated for $R_{\text{eff}}(t) < 1$ under that specific rate of infection. However, these numbers should be interpreted with caution; the low values for δ are in part due to the social restrictions at the time. The gradual relaxation in social restrictions may have contributed to the increases in δ, and therefore we have to consider this when interpreting the numbers. This means that a 71% triple vaccination rate may have suppressed the outbreak in Western Australia, but it would have to have been combined with similarly strict social restrictions as we saw in the early stages of the outbreak. This may not be the preferred control method by policy makers given the lower severity of the Omicron strain in comparison to previous strains, however it is important to note for any new strains that may arise in the near future. As shown for $\delta = 0.905$, no level of triple vaccination is enough to suppress the outbreak. Hence a certain level of vaccinations alone would have not been enough to suppress the Omicron outbreak, and therefore should not have been the sole consideration in opening the borders.

If we choose to remove the assumption that people are either unvaccinated or fully (triple) vaccinated, we have a greater range of possible solutions for $\hat{V}_m$, depending on the δ we set. For example, for the $\delta = 0.442$ case, we have a possible solution[3] with $\hat{V}_0 = 5\%, \hat{V}_1 = 21\%, \hat{V}_2 = 18\%, \hat{V}_3 = 56\%$. However, for $\delta = 0.905$, there is still no possible combination that suppresses the spread of COVID-19 in Western Australia.

4 WA TOWNS DATASET

Below is the dataset detailing the town population, location and which region the town belongs to, for all WA towns used. Color coding is used based on the colors shown in the coloured transport network in the main text.

[3] Note that a lower limit was set on the number of unvaccinated people never falling below 5%.

No.	Town	Pop.	Long.	Lat.	Region
1	Perth	1874578	115.9	-32	Perth
2	Mandurah	80813	115.7	-32.5	Peel
3	Bunbury	71090	115.6	-33.3	South West
4	Geraldton	31982	114.6	-28.8	Mid West
5	Kalgoorlie-Boulder	29875	121.5	-30.7	Goldfields-Esperance
6	Albany	29373	117.9	-35	Great Southern
7	Busselton	25329	115.3	-33.7	South West
8	Karratha	15828	116.8	-20.7	Pilbara
9	Broome	13984	122.2	-18	Kimberley
10	Port Hedland	13828	118.5	-21	Pilbara
11	Esperance	10421	122	-33.5	Goldfields-Esperance
12	Collie	7192	116.2	-33.3	South West
13	Northam	6548	116.7	-31.6	Wheatbelt
14	Margaret River	6392	115.2	-34.2	South West
15	Dunsborough	6039	115.1	-33.6	South West
16	Newman	4567	119.7	-23.4	Pilbara
17	Carnarvon	4426	113.7	-24.9	Gascoyne
18	Narrogin	4274	117.2	-32.9	Wheatbelt
19	Manjimup	4213	116.2	-34.5	South West
20	Katanning	3702	117.7	-33.6	Great Southern
21	Tom Price	2956	117.8	-22.7	Pilbara
22	Port Denison-Dongara	2782	114.9	-29.2	Mid West
23	Harvey	2750	115.9	-33.1	South West
24	Merredin	2636	118.4	-31.6	Wheatbelt
25	Denmark	2558	117.1	-34.9	Great Southern
26	York	2548	116.8	-31.9	Wheatbelt
27	Donnybrook	2520	115.8	-33.6	South West
28	Exmouth	2486	114.1	-21.9	Gascoyne
29	Waroona	2397	115.9	-32.9	Peel
30	Capel	2020	115.6	-33.5	South West
31	Mount Barker	1905	117.7	-35.1	Great Southern
32	Kambalda West	1789	121.7	-31.2	Goldfields-Esperance
33	Little Grove	1703	117.9	-35.1	Great Southern
34	Moora	1577	116.1	-30.6	Wheatbelt
35	Wickham	1572	117.1	-20.7	Pilbara
36	Cowaramup	1546	115.1	-33.9	South West
37	Halls Creek	1546	127.7	-18.2	Kimberley
38	Jurien Bay	1425	115.1	-30.3	Wheatbelt
39	Paraburdoo	1359	117.7	-23.2	Pilbara
40	Wagin	1358	117.3	-33.3	Wheatbelt
41	Kalbarri	1349	114.2	-27.7	Mid West
42	Kojonup	1165	117.2	-33.8	Great Southern
43	Fitzroy Crossing	1141	125.6	-18.2	Kimberley
44	Augusta	1109	115.2	-34.3	South West

Table S3. Western Australian towns used in this study, sorted by population.

REFERENCES

Pulliam JR, van Schalkwyk C, Govender N, von Gottberg A, Cohen C, Groome MJ, et al. Increased risk of SARS-CoV-2 reinfection associated with emergence of the Omicron variant in South Africa. *MedRxiv* (2021).

Altarawneh HN, Chemaitelly H, Hasan MR, Ayoub HH, Qassim S, AlMukdad S, et al. Protection against the Omicron variant from previous SARS-CoV-2 Infection. *New England Journal of Medicine* **386** (2022) 1288–1290. doi:10.1056/NEJMc2200133.

[Dataset] Turner R. First reports of reinfections raise questions over WA COVID recovery timeline (May 21, 2022). Available online: `https://www.abc.net.au/news/2022-05-21/first-reports-of-covid-reinfections-in-wa-emerge/101086314` (accessed on 23 May 2022).

Berman Y. *Modelling COVID-19 Transmission in Western Australia*. Master's thesis, Department of Mathematics and Statistics (May 27, 2022). Honours Thesis presented for the Bachelor of Philosophy (Honours) degree of the University of Western Australia.

[Dataset] Covid Live. WA (2022). Available online: `https://covidlive.com.au/wa` (accessed on 16 May 2022).

Zhao S, Tang B, Musa SS, Ma S, Zhang J, Zeng M, et al. Estimating the generation interval and inferring the latent period of COVID-19 from the contact tracing data. *Epidemics* **36** (2021) 100482. doi:https://doi.org/10.1016/j.epidem.2021.100482.

Xin H, Li Y, Wu P, Li Z, Lau EHY, Qin Y, et al. Estimating the Latent Period of Coronavirus Disease 2019 (COVID-19). *Clinical Infectious Diseases* **74** (2021) 1678–1681. doi:10.1093/cid/ciab746.

[Dataset] World Health Organisation. Contact tracing and quarantine in the context of the Omicron SARS-CoV-2 variant (February 17, 2022). Available online: `https://www.who.int/publications/i/item/WHO-2019-nCoV-Contact-tracing-and-quarantine-Omicron-variant-2022.1` (accessed on 1 May 2022).

Department of Health, Government of Western Australia. COVID-19 close contacts. *HealthyWA* (May 6, 2022). `https://www.healthywa.wa.gov.au/Articles/A_E/Coronavirus/COVID19-close-contacts#:~:text=If%20you%20have%20recovered%20from,a%20close%20contact%20and%20you` (accessed 10 May 2022).

Andrews N, Stowe J, Kirsebom F, Toffa S, Rickeard T, Gallagher E, et al. COVID-19 Vaccine Effectiveness against the Omicron (B.1.1.529) Variant. *New England Journal of Medicine* **386** (2022) 1532–1546. doi:10.1056/NEJMoa2119451.

Virtanen P, Gommers R, Oliphant TE, Haberland M, Reddy T, Cournapeau D, et al. SciPy 1.0: Fundamental Algorithms for Scientific Computing in Python. *Nature Methods* **17** (2020) 261–272. doi:10.1038/s41592-019-0686-2.

Walker DM, Mees AI. Noise Reduction of Chaotic Systems by Kalman filtering and by Shadowing. *International Journal of Bifurcation and Chaos in Applied Sciences and Engineering* **7** (1996) 769–779.

Anderson BDO. *Optimal Filtering*. Dover Books on Electrical Engineering (Newburyport: Dover Publications) (2012).

[Dataset] Kagi J. WA's COVID-19 border will stay shut indefinitely. What now, and when might the state reopen? (January 21, 2022). Available online: `https://www.abc.net.au/news/2022-01-21/wa-border-opening-delayed-what-happens-now-explainer/100772052` (accessed on 18 May 2022).

[Dataset] Marcus L. Western Australia ends one of the world's longest border closures (March 3, 2022). https://edition.cnn.com/travel/article/western-australia-border-reopening-intl-hnk/index.html, (accessed 1 May 2022).

Delamater PL, Street EJ, Leslie TF, Yang YT, Jacobsen KH. Complexity of the Basic Reproduction Number (R0). *Emerging infectious diseases* **25** (2019) 1–4.
